# Ratiometric fluorescent test paper based on silicon nanocrystals and carbon dots for sensitive determination of mercuric ions

**DOI:** 10.1098/rsos.171922

**Published:** 2018-06-27

**Authors:** Xinfeng Guo, Cui Liu, Nian Li, Shudong Zhang, Zhenyang Wang

**Affiliations:** 1 School of Electronic and Electrical Engineering, Nanyang Institute of Technology, Nanyang 473004, Henan, People's Republic of China; 2 Institute of Intelligent Machines, Chinese Academy of Sciences, Hefei 230031, Anhui, People's Republic of China

**Keywords:** carbon dots, ratiometric fluorescent, test paper

## Abstract

Classical dye absorption-based pH test papers are widely used to measure the degree of acidity of media for quantification or semi-quantification by the observation of the naked eye on the variation of colour. However, it remains a challenging task to extend portable and cheap methods to a wide range of analytes for accurate quantification. Here, we report a dosage-sensitive test paper for the assay of mercury ions (Hg^2+^) with wide colour evolution. The ratiometric fluorescent probe was prepared by mixing blue-emission silicon nanocrystals (Si NCs) and red-emission carbon dots (r-CDs). The Si NCs serve as the reaction site and the r-CDs as the internal reference. Upon the addition of Hg^2+^, the blue fluorescence of the Si NCs was quenched, while the red fluorescence of the r-CDs remained constant, resulting in the consecutive fluorescence colour changes from blue to orange red. The probe demonstrates high selectivity and sensitivity of the visualization assay of Hg^2+^ with a detection limit of 7.63 nM. Moreover, we printed the ratiometric fluorescence probe result onto a piece of filter paper to prepare a test paper, which represents an on-site, sensitive, rapid and cost-effective method for the visual determination of Hg^2+^ by the naked eye.

## 1. Introduction

Heavy metal pollution has always been a serious environmental concern across the world for decades, being widely distributed in air, water, food and soil. In particular, mercury is a toxic heavy metal, and it can cause permanent damage to the human body even at a very low concentration [1–2]. People are put on high alert to avoid the occurrence of shocking pollution events. Therefore, the detection of mercury ions as a source of pollution is necessary by governments or societal institutions and the maximum allowable level of mercuric ions has been set to 10 nM by the US Environmental Protection Agency [3]. Currently, the widely used methods for the determination of mercuric ions include atomic absorption spectroscopy [4], inductively coupled plasma mass spectroscopy [5], X-ray fluorescence spectrometry [6] and anodic stripping voltammetry [7]. Although these assays are of high sensitivity and selectivity, they are usually expensive and time-consuming, and require tedious sample pretreatments and expensive equipment operated by well-trained technicians. Thus, there is a great demand to develop a fast, sensitive and reliable assay of Hg^2+^ to improve food safety and environmental protection.

Fluorescent test paper obtained by printing fluorescent probes onto paper-based substrates for visualization assay has attracted gradually increasing attention in recent years [8–10], owing to its unique advantages of portable feasibility, low cost and easy operation. Most fluorescent test papers contained a single fluorescent sensor and were constructed on the fluorescent ‘turn on' [11] or ‘turn off' [12] mode, which may cause interferences by excess analyte or other environmental factors, consequently reducing the sensitivity and limiting their quantitative capability in practical applications. A ratiometric fluorescent sensor possessing a few individual fluorescence emission peaks could greatly reduce the above limitations by self-calibration, resulting in colour change of the dual-emission fluorescent sensor and easy recognition by the naked eye under a portable UV lamp.

The materials of the assay of Hg^2+^ included quantum dots (QDs) [12–14], fluorescent dye derivatives [15], plasmonic metal nanoparticles [16], up-conversion nanoparticles [17], carbon dots (CDs) [18,19] and silicon nanocrystals (Si NCs) [20], and exhibited good potential in the application to detect Hg^2+^. However, QDs have the limitations of well-known toxicity and potential environmental hazard of the heavy metals, the fluorescent dyes suffer from photobeaching, and plasmonic metal nanoparticles are usually Ag and Au, which are expensive. CDs are novel fluorescent nanomaterials to which has been paid more and more attention because of their high quantum yield, particular fluorescent properties, good photostability, water solubility, lower cytotoxicity and excellent biocompatibility [21]. In addition, Si NCs are also attractive due to their favourable biocompatibility and low cytotoxicity [22]. Herein, we propose a ratiometric fluorescent sensor by mixing Si NCs with red-emission carbon dots (r-CDs) for the detection of Hg^2+^. Upon the addition of Hg^2+^, the blue fluorescence of Si NCs was quenched, while the red fluorescence of the r-CDs remained constant. With the aid of r-CDs, the ratiometric fluorescent sensor displayed discernable colour evolution from blue to purple to pink to orange red with increasing concentration of Hg^2+^. The probe demonstrates high selectivity and sensitivity of the visualization assay of Hg^2+^, with a detection limit of 7.63 nM. Based on this principle, the ratiometric fluorescent sensor contained a mixture of r-CDs and Si NCs with a fluorescent ratio of 1 : 7, printed onto a piece of paper to construct ratiometric fluorescent test papers. This kind of test paper offers great potential for the detection of Hg^2+^ in tap and lake water.

## 2. Material and methods

### 2.1. Regents and instruments

*p*-Phenylenediamine (p-PDA) and (3-aminopropyl)trimethoxysilane (APTMS) were purchased from Aldrich. Sodium citrate, NaOH, H_2_SO_4_, ethanol and all the metal salts were supplied by Sinopharm Chemical Reagent Company, Ltd (Shanghai, China). All chemicals were used as received without further purification unless otherwise specified. Ultrapure water (18.2 MΩ cm) was prepared with a Millipore water purification system.

The structure and morphology were examined using a JEOL 2010 transmission electron microscope. Fluorescence spectra were recorded with a Cary Eclipse fluorescent spectrophotometer. The UV–visible absorption spectra were obtained with a Shimadzu UV-2550 spectrometer. Fourier transform infrared (FT-IR) spectra were obtained with a Thermo Fisher Nicolet iS10 FT-IR spectrometer. X-ray photoelectron spectroscopy (XPS) measurements were carried out with a Thermo ESCALAB 250 high performance electron spectrometer with Al K*α* (1486.6 eV) radiation. Fluorescent photos were taken under an AGL-9406 portable UV lamp (365 nm) by a Canon 350 D digital camera.

### 2.2. Synthesis of silicon nanocrystals

Fluorescent Si NCs were synthesized according to a reported method with minor modification [20]. Briefly, 1 g of sodium citrate was dissolved in 30 ml of water and then stirred under bubbling nitrogen for 30 min. A 3 ml aliquot of APTMS was added and homogeneously agitated, then the solution was transferred into poly(tetrafluoroethylene)-lined autoclaves. After heating at 170°C for 12 h and cooling down to room temperature naturally, the resultant transparent solution was purified by dialysis using a membrane with a molecular weight cut-off of 1 kDa for 12 h. Finally, the solution was collected and stored at 4°C for use.

### 2.3. Synthesis of red-emission carbon dots

The r-CDs were prepared by a modified hydrothermal method according to a reported method with minor modification [23,24]. An amount of 0.5 g of p-PDA was dissolved in 50 ml of ethanol, and the solution was subsequently transferred into poly(tetrafluoroethylene)-lined autoclaves. After heating at 200°C for 12 h and cooling down to room temperature naturally, the obtained products were purified with silica column chromatography using ethyl acetate as the eluent. Finally, the obtained solution was dried by a rotary evaporator, and the purified r-CDs were redispersed in 50 ml of ultrapure water for further use.

### 2.4. Detections of Hg^2+^ ions

The ratiometric fluorescent probe was prepared by mixing the Si NCs and r-CDs with the fluorescence intensity ratio of 1 : 7 in 2 ml of phosphate buffer (pH 5.9). Different concentrations of Hg^2+^ ions were added into the ratiometric fluorescent probe solution and reacted for 3 min; the fluorescent spectra were recorded with a fluorescence spectrometer.

### 2.5. Preparation of test papers

A commercial ink cartridge was washed with ultrapure water until the ink powder was cleared out completely, and dried in an oven at 70°C for 3 h. Then, ‘ink' containing the mixed solution of Si NCs and r-CDs (fluorescence intensity ratio of 7 : 1) was injected into the vacant cartridge. A filter paper was stuck onto a piece of A4 paper. The ‘ink' was printed on the filter paper through an inkjet printer connected with a computer, and the printing process was repeated 20 times. Finally, the filter paper displayed a strong blue fluorescent colour under a 365 nm UV lamp.

## 3. Results and discussion

**Figure 1.**
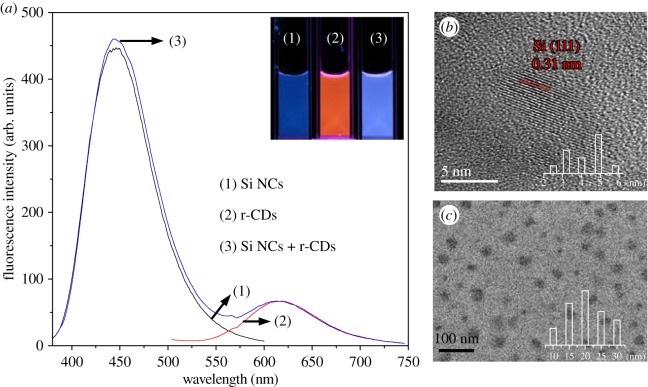
(*a*) Fluorescence spectra of blue emission of Si NCs (1), red emission of CDs (2) and the mixed sensory system (3) at the excitation of 350 nm; the inset photos were taken under a 365 nm UV lamp. TEM images of (*b*) Si NCs and (*c*) r-CDs; the insets are the size distributions of Si NCs and r-CDs.

The Si NCs and r-CDs were prepared using APTMS and p-PDA as starting materials by the hydrothermal and solvothermal methods, respectively. The properties of the Si NCs and r-CDs were characterized by fluorescence spectroscopy, FT-IR spectra and transmission electron microscopy (TEM). As shown in figure 1*a*, the maximum emission peaks of Si NCs and r-CDs are at 445 nm and 615 nm, meaning that the Si NCs emit blue fluorescence, while the r-CDs display red fluorescence under a 365 nm UV lamp. Thus, the Si NCs and r-CDs system is a mixture emitting strong blue fluorescence. The fluorescence spectra of Si NCs and r-CDs were further recorded every 5 min for 1 h irradiated using a 365 nm UV lamp. Clearly, the fluorescence intensities exhibited no distinct change, implying that both the Si NCs and r-CDs exhibit good stability against photobleaching (electronic supplementary material, figure S1). The TEM image of the Si NCs (electronic supplementary material, figure S2) revealed spherical nanoparticles with a diameter of 3–5 nm. A higher magnification image of a single Si NC (figure 1*b*) clearly revealed the interplanar spacing of 0.31 nm corresponding to the (111) lattice planes of Si [19,20], while the as-prepared r-CDs possessed a good monodispersity with a diameter of approximately 20 nm (figure 1*c*). Additionally, the FT-IR spectrum of the Si NCs showed peaks at 1585 and 3410 cm^−1^, assigned to N–H bending vibration and stretching vibration (electronic supplementary material, figure S3). The result indicated the existence of the amide groups on the surface of Si NCs [20,24,25]. It could also be supported by the XPS results (electronic supplementary material, figure S4); the N1s band at 399.3 eV is associated with amino N [24,25].

**Scheme 1.**
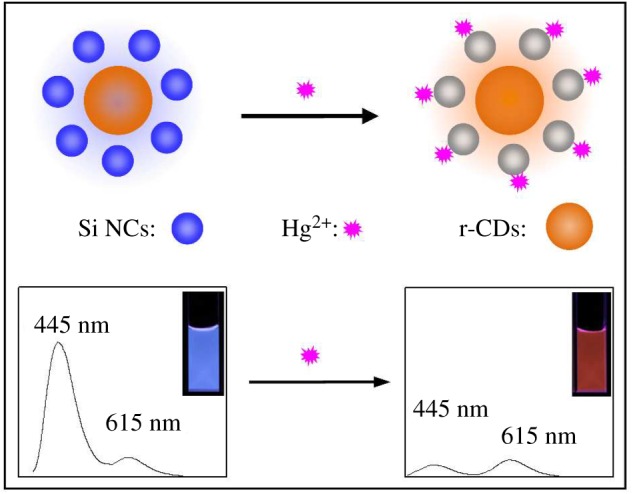
Scheme illustration of the sensory system for the visual assay of Hg^2+^. The blue fluorescence of Si NCs is quenched by Hg^2+^, while the red fluorescence of r-CDs remains stable with the addition of Hg^2+^, resulting in the colour evolution from blue to orange red.

For r-CDs, there are large amounts of residue of p-PDA ligands at the surface of the r-CDs, which we have confirmed in our paper [10] and the FT-IR spectrum of the r-CDs (electronic supplementary material, figure S5). Meanwhile, we have shown that the obtained r-CDs were insensitive to Hg^2+^ (electronic supplementary material, figure S6) [9,10]. However, the Si NCs were modified with the amide groups to obtain the supersensitivity to Hg^2+^ for the quenching of blue fluorescence [20]. The Si NCs could be serving as a reaction site for Hg^2+^, by which the blue fluorescence was effectively quenched by Hg^2+^. According to Yu's report, this is probably due to the interaction of Si NCs with Hg^2+^ derived from amino groups on the surfaces of Si NCs via the affinity of Hg^2+^ to N, and the fluorescence quenching of Si NCs by Hg^2+^ involves both dynamic and static processes [20]. Therefore, as shown in scheme 1, the ratiometric fluorescent probe was prepared by mixing the blue-emission Si NCs and red-emission r-CDs. The ratiometric fluorescent probe emitted a strong blue fluorescence, and the fluorescence spectrum displayed two emission bands at 445 and 615 nm. The r-CDs could be the internal standard in the ratiometric fluorescence system due to their stability against Hg^2+^. Upon exposure to Hg^2+^, an obvious wide-range colour evolution can be observed under a UV lamp, which can be applied for the visual assay of Hg^2+^. Additionally, the photostability of the ratiometric probe was studied; this demonstrated its excellent photostability (electronic supplementary material, figure S7).

**Figure 2.**
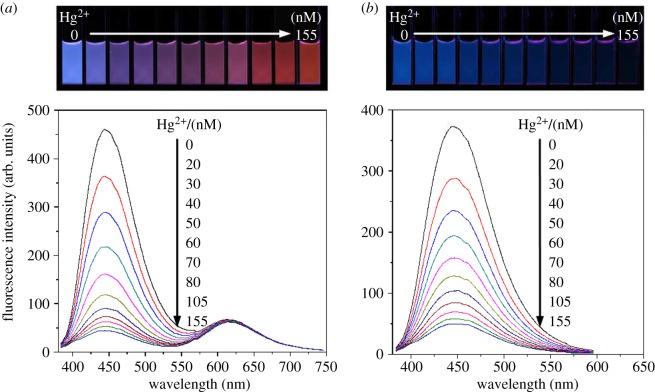
The fluorescence spectra of (*a*) the ratiometric fluorescent probe and (*b*) the blue Si NCs in the presence of different concentrations of Hg^2+^. Inset images correspond to the fluorescence photos under a 365 nm UV lamp.

The dosage responses of the ratiometric probe and blue Si NCs to Hg^2+^ have been examined. As can be seen in figure 2*a*, the fluorescence at 445 nm from the Si NCs was continuously quenched, whereas the peak at 615 nm originated from r-CDs remained highly steady upon the addition of Hg^2+^, leading to distinguishable colour variations from blue to purple to pink to orange red under a 365 nm UV lamp. On the other hand, the fluorescence intensity of the blue Si NCs decreased accordingly, and the colour became weak with the addition of Hg^2+^. A which was difficult to distinguish by the naked eye (figure 2*b*). These results revealed that the ratiometric fluorescent probe is available for the visual detection of Hg^2+^ by the naked eye. The detailed experiments displayed that the widest and consecutive colour evolution could be obtained at the 7 : 1 ratio for Si NCs and r-CDs in fluorescence intensity (electronic supplementary material, figure S8). The dynamics experiments exhibited that the fluorescent response to Hg^2+^ was completed in less than 5 min (electronic supplementary material, figure S9), indicating that the fluorescence quenching of the ratiometric probe by Hg^2+^ is a quick process. Moreover, we also studied the effect of phosphate buffer pH on the detection of Hg^2+^ of the ratiometric probe. The results show that the maximum fluorescence quenching occurs at pH 5.9 (electronic supplementary material, figure S10). The fluorescence intensity ratio (*I*_445_/*I*_615_) of ratiometric fluorescence probe decreased proportionately with the addition of Hg^2+^. A relationship can be set up between the concentration of the Hg^2+^ and *I*_445_/*I*_615_. A good linear relationship (*I*_445_/*I*_615 _= −0.011 × [Hg^2+^] + 0.840) was obtained with a correlation coefficient *R*^2^ of 0.995 for the concentration of Hg^2+^ ranging from 0 to 105 nM (electronic supplementary material, figure S11), and the detection limit (sensitivity) was defined to be three times the standard deviation of background (3*σ*), which was calculated to be 7.63 nM.

**Figure 3.**
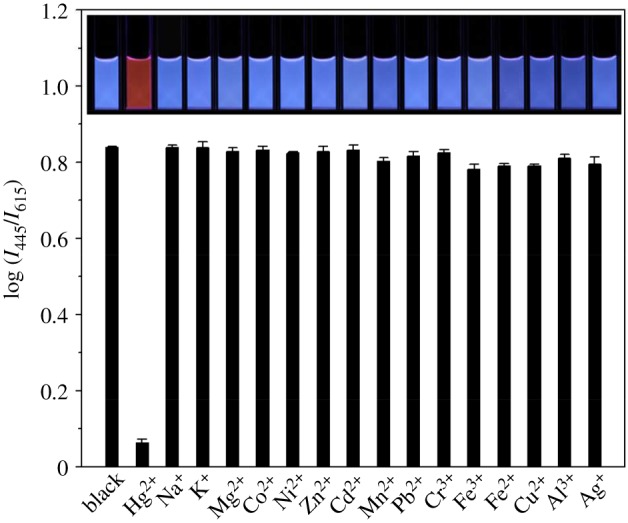
The fluorescence response of selectivity to different amounts of Hg^2+^. The selectivity data were obtained using the *I*_445_/*I*_615_ ratio of the mixing the Si NCs and r-CDs (7:1 in fluorescence intensity) in phosphate buffer (pH = 5.9) with the addition of 105 nM Hg^2+^. The inset images show the corresponding fluorescent photos under a 365 nm UV lamp.

To study the selectivity of the ratiometric fluorescence probe for Hg^2+^, the fluorescence intensity ratio (*I*_445_/*I*_615_) was investigated at the same conditions with the addition of a variety of metal ions. As shown in figure 3, the fluorescence intensity ratio (*I*_445_/*I*_615_) of the ratiometric fluorescence probe was quenched by about 92.5% on adding 105 nM Hg^2+^, and the fluorescence colour changes from blue to orange red under a 365 nm UV lamp. However, with adding 2 µM of other metal ions (Na^+^, K^+^, Mg^2+^, Co^2+^, Ni^2+^, Zn^2+^, Cd^2+^, Mn^2+^, Pb^2+^, Cr^3+^, Fe^3+^, Fe^2+^, Cu^2+^ Al^3+^, Ag^+^) into the ratiometric fluorescence system, almost no obvious fluorescence intensity ratio and colour changes were observed (inset image of figure 3), which indicated the excellent selectivity of the ratiometric fluorescence probe to Hg^2+^ over other metal ions. The anti-interference ability of the ratiometric fluorescence probe system with other metal ions was also investigated (electronic supplementary material, figure S12). On adding 105 nM Hg^2+^ in the ratiometric fluorescence probe system, that is when 10-fold excess of the interfering ions was added, it has a negligible interfering effect on Hg^2+^ determination, indicating that the method has good selectivity and colour variations to Hg^2+^.

To test the reliability of the ratiometric fluorescence probe system, we added different amounts of Hg^2+^ into real water samples, including tap water and lake water. All the real water samples were first filtered through a filter paper to remove any impurities. Then we spiked four different amounts of Hg^2+^ into the water samples. As shown in electronic supplementary material, table S1, the recovery tests and the relative standard deviations of each concentration were studied in triplicate and the averages are presented with standard deviations. The recoveries of Hg^2+^ in tap water and lake water were in the range from 99.5% to 105.3% and from 98.0% to 103.0%, respectively (electronic supplementary material, table S1), demonstrating that the ratiometric fluorescence probe system can efficiently work for the detection of Hg^2+^ in real water samples.

**Figure 4.**
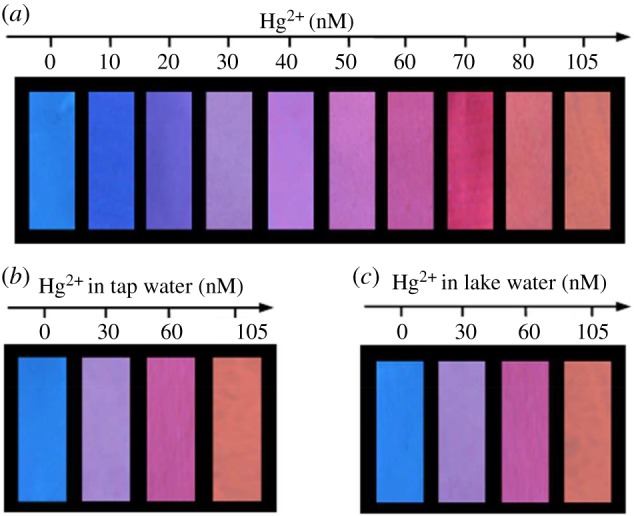
(*a*) The visual detection of Hg^2+^ using the fluorescent test papers prepared by printing ratiometric fluorescence probe results with ink onto a piece of filter paper. (*b,c*) The visual detection of Hg^2+^ in tap water and lake water, respectively. The photos were taken under a 365 nm UV lamp.

To realize the rapid and on-site detection of Hg^2+^, fluorescent test paper was prepared for the visual determination of Hg^2+^. The ratiometric fluorescent probe used fluorescent ink, and the result was printed onto a piece of filter paper through an inkjet printer connected with a computer; the printing process was repeated 20 times. The filter paper was non-fluorescent to avoid the interference to visualization colorimetry, and the as-prepared test papers exhibited blue fluorescence under a 365 nm UV lamp. When dropping Hg^2+^ solution onto the prepared test paper, the fluorescence colour of the test paper exhibited a distinct colour change from blue to purple to pink to orange red by the naked eye under a 365 nm UV lamp (figure 4*a*). The visualization of the test paper for the detection of Hg^2+^ was also studied by spiking different amounts of Hg^2+^ into real water samples. The fluorescence colours of test paper were almost the same as that in tap water (figure 4*b*) when spiked with Hg^2+^ by the concentrations of 0, 30, 60 and 105 nM into the lake water samples (figure 4*c*), respectively, corresponding to the colours in figure 4*a*. Therefore, the ratiometric fluorescent test papers in our work are suitable for the visual determination of Hg^2+^ ions in real water samples.

## 4. Conclusion

In conclusion, we designed a strategy to construct a ratiometric fluorescence probe and a paper-based sensor for the determination of Hg^2+^ by a dosage-sensitive colour evolution. The probe was prepared by mixing blue emission Si NCs with orange red emission r-CDs, based on the fact that the blue fluorescence of Si NCs was quenched by Hg^2+^, while the r-CDs remained with no change, resulting in the consecutive fluorescence colour changes from blue to orange red upon the addition of Hg^2+^. The probe demonstrates high selectivity and sensitivity of visual detection with a detection limit of 7.63 nM in aqueous solution. Moreover, we printed the ratiometric fluorescence probe result onto a piece of filter paper to prepare a test paper, which represents an on-site, sensitive, rapid and cost-effective method for the visual determination of Hg^2+^ by the naked eye. The results reported here have revealed that the method could be extended to apply in visual analysis in the environmental, biological and food fields.

## Supplementary Material

Ratiometric Fluorescent Test Paper Based on Silicon Nanocrystals and Carbon Dots for Sensitive Determination of Mercuric ions

## Data accessibility

Some measurement information is presented in the electronic supplementary material of this article.
