# Supplementary material for: Ratiometric fluorescent test paper based on silicon nanocrystals and carbon dots for sensitive determination of mercuric ions
Source: R Soc Open Sci. 2018 Jun 27;5(6):171922. doi: 10.1098/rsos.171922 (PMC6030300; doi:10.1098/rsos.171922)
Supplement: Ratiometric Fluorescent Test Paper Based on Silicon Nanocrystals and Carbon Dots for Sensitive Determination of Mercuric ions [file rsos171922supp1.docx]

**Ratiometric Fluorescent Test Paper Based on Silicon Nanocrystals and Carbon Dots for Sensitive Determination of Mercuric ions**

**Xinfeng Guo,^1^ Cui Liu,*^2^ Nian Li,^2^ Shudong Zhang*^2^, and Zhenyang Wang^2^**

^1^ School of Electronic and Electrical Engineering, Nanyang Institute of Technology, Nanyang 473004, Henan, P. R. China.

^2^ Address here Institute of Intelligent Machines, Chinese Academy of Sciences, Hefei, Anhui, 230031, P. R. China.

E-mail: cliu0724@mail.ustc.edu.cn, sdzhang@iim.ac.cn.

**Figure S1:** Photostability of the Si NCs and r-CDs in 60 min.


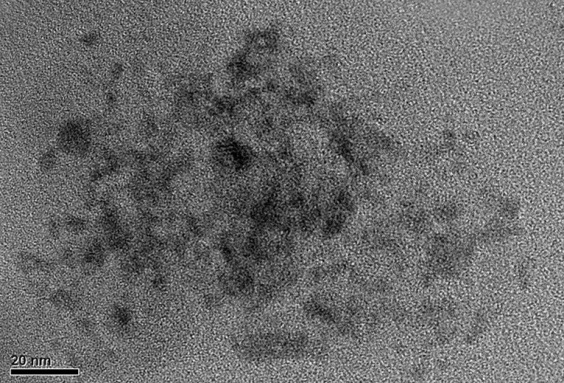


**Figure S2:** TEM image of the Si NCs.

**Figure S3:** The FT-IR spectrum of the Si NCs.

**Figure S4:** The XPS N1s spectrum of the Si NCs.

**Figure S5:** The FT-IR spectrum of the r-CDs.

**Figure S6:** The fluorescence spectra of the r-CDs in the presence of different concentration of Hg^2+^. The inset images corresponded to fluorescence photos under a 365 nm UV lamp.

**Figure S7:** Photostability of the ratiometric probe in 120 min.

**Figure S8:**The fluorescent spectra of mixture of Si NCs and r-CDs at different ratios with the addition of Hg^2+^. The ratios of fluorescent intensity (blue to red) were (A) 3:1, (B) 5:1, (C) 7:1, (D) 9:1. The insets show the corresponding fluorescent photos under a 365 nm UV lamp.

**Figure S9:** The dynamics of fluorescent responses of ratiomertric fluorescence (I_445_/I_615_) to 105 nM Hg^2+^.

**Figure S10:** The effect of phosphate buffer pH on the detection of Hg^2+^.

**Figure S11** The fluorescence intensity ratio (I_445_/I_615_) of the ratiometric fluorescence probe versus the concentration of Hg^2+^.

**Figure S12** The anti-interference tests were performed by the coexistence of an excess of interfering ions (2 μM).

**Table S1.** The recoveries of Hg^2+^ in tap water and lake water using the fluorescent measurements of ratiometric fluorescence probe.

| **Spiked**  **concentration**  **(nM)** | **Tap water** | | |  | **Lake water** | | |
| --- | --- | --- | --- | --- | --- | --- | --- |
|  | **Found**  **(nM)** | **Recovery**  **(%)** | **RSD**  **(%)** |  | **Found**  **(nM)** | **Recovery**  **(%)** | **RSD**  **(%)** |
| 20 | 20.1 | 100.5 | 3.4 |  | 21.2 | 106 | 2.3 |
| 40 | 39.5 | 98.8 | 2.6 |  | 40.5 | 101.2 | 5.3 |
| 60 | 63.2 | 105.3 | 5.4 |  | 58.8 | 98.0 | 6.9 |
| 80 | 79.6 | 99.5 | 7.1 |  | 82.4 | 103.0 | 5.9 |
